# Comparing the Safety and Efficacy of Proton Pump Inhibitors and Histamine-2 Receptor Antagonists in the Management of Patients With Peptic Ulcer Disease: A Systematic Review

**DOI:** 10.7759/cureus.44341

**Published:** 2023-08-29

**Authors:** Maha Begg, Mawada Tarhuni, Monique N. Fotso, Natalie A Gonzalez, Raghavendra R Sanivarapu, Usama Osman, Abishek Latha Kumar, Aishwarya Sadagopan, Anas Mahmoud, Safeera Khan

**Affiliations:** 1 Internal Medicine, California Institute of Behavioral Neurosciences & Psychology, Fairfield, USA; 2 Internal Medicine, California Institute of Behavioral Neurosciences & Psychology, Fairfiled, USA; 3 Internal Medicine, Pediatrics, California Institute of Behavioral Neurosciences & Psychology, Fairfield, USA

**Keywords:** h2 receptor antagonist, drug dosage, safety and efficacy, peptic ulcer disease, proton pump inhibitors

## Abstract

Peptic ulcer disease (PUD) refers to the occurrence of an open erosion in the inner lining of the stomach, duodenum, or sometimes lower esophagus. Treatments like proton pump inhibitors (PPIs) or histamine 2 receptor antagonists (H2RAs) are available on the market to efficiently treat the break in the mucosal lining. However, there is little evidence about the effects of the medication on the type and location of the ulcer and the epigastric pain caused by disintegration and increased acidity in the stomach.

Given the above, we conducted a systematic review comparing the safety and efficacy of PPIs and H2RAs in various ulcer locations (gastric, duodenal, and pre-pyloric) and the effect of prolonging the treatment with the same medication or changing into a drug from another class in treatment-resistant ulcers. We employed major research literature databases and search engines such as PubMed, Medical Literature Analysis and Retrieval System Online (MEDLINE), Science Direct, and Google Scholar to find relevant articles. After a thorough screening, a quality check using various tools, and applying filters that suited our eligibility criteria, we identified eight articles, of which five were random clinical trials (RCTs), two review articles, and one meta-analysis. This study compares the different side effects of PPIs and H2RAs. Most studies concluded that omeprazole is superior in healing ulcers and bringing pain relief and that patients resistant to H2RAs can be treated better when switched to a PPI. This study also discusses the adverse effects of chronic use, such as diarrhea, constipation, headaches, and gastrointestinal infections. Patients on long-term PPI therapy are required to take calcium supplements to prevent the risk of fractures in older adults. Regarding long-term outcomes, PPIs remain the mainstay of treatment for peptic ulcer disease, based on the papers we reviewed.

## Introduction and background

Peptic ulcers are open sores in the stomach and duodenum due to decreased mucosal protection against gastric acid. Peptic ulcer disease (PUD) symptoms may include abdominal pain, bloating, heartburn, nausea, vomiting, weight loss, bleeding, or perforation [1]. In the United States, self-reported, physician-diagnosed peptic ulcer disease was 10% in 1990, and the approximate incidence has now increased to 500,000 new cases per year, affecting both sexes equally; however, a lower risk is found among Blacks and Hispanics. [2]. Gastroprotection drugs, such as proton-pump inhibitors (PPIs) and histamine-2 receptor antagonists (H2RAs), have been developed to protect and heal damaged mucosa and alleviate its associated symptoms [3].

Since the introduction of omeprazole in 1989, PPIs have progressively become the mainstay in treating acid-related disorders [4]. They work by undergoing acidic activation within the parietal cells to allow the PPI to be ionized and form covalent disulfide bonds with cysteines of the H(+)-K(+)-adenosine triphosphatase (H(+)-K(+)-ATPase). Once the PPI binds to the proton pump, the pump is inactivated, preventing the release of H(+) ions. The effectiveness of PPIs comes from its structure [5]. The H2RAs decrease the secretion of H(+) ions by parietal cells.

Peptic ulcer disease usually occurs in the stomach and proximal portion of the duodenum. Gastric ulcers occur primarily due to the widespread use of low-dose aspirin and nonsteroidal anti-inflammatory drugs (NSAIDs) in treating chronic diseases such as rheumatoid arthritis and the prevention of cardiovascular disease and stroke [6], which prevent the release of prostaglandins that offer protective layering to the stomach wall. Therefore, the decrease in mucosal protection against gastric acid predisposes the stomach lumen to the harsh effects of the acid. Duodenal ulcers are often caused by Zollinger-Ellison syndrome (ZES), a clinical syndrome characterized by excessive gastric acid production. This occurs due to the ectopic secretion of gastrin by a neuroendocrine tumor called a gastrinoma. These tumors are commonly found in the duodenum and pancreas. [7] Currently, *Helicobacter pylori* (*H. pylori*) is one of the major risk factors for duodenal ulcers, favoring mostly those who are socioeconomically disadvantaged [2]. Other causes of PUD include the use of antiplatelet drugs, stress, cytomegalovirus infections, Behçet's disease, Crohn's disease, and end-stage liver disease [6]. The primary complications of PUD are bleeding, perforation, penetration, and obstruction. Among these complications, bleeding is most likely to occur, and its incidence continues to rise, requiring urgent surgical attention [8].

Clinical guidelines recommend PPIs as first-choice gastroprotection drugs, supported by systematic reviews and meta-analyses in clinical settings [9] [3]. Still, to date, no comprehensive effort has been made to compare the effectiveness of PPIs and H2RAs in the management of treatment-resistant or refractory peptic ulcers.

## Review

Methods

We conducted our systematic review and reported the results according to the Preferred Reporting Items for Systematic Reviews and Meta-Analysis (PRISMA) guidelines [10].

Search Strategy

We used electronic databases PubMed, Medical Literature Analysis and Retrieval System Online (MEDLINE), ScienceDirect, and Google Scholar to identify the relevant articles using Medical Subject Headings (MeSH) and keywords. The keywords included "peptic ulcer disease," "proton pump inhibitors," and "H2 receptor blocker". We used the Boolean method to assemble the keywords for an algorithm to use in PubMed. The articles were filtered to highlight those most relevant to our research topic.

The MeSH strategy for PubMed, PMC, and Medline is as follows:

("Peptic Ulcer/drug therapy"(Majr) OR "Peptic Ulcer/prevention and control"(Majr) OR "Peptic Ulcer/therapy"(Majr)) AND ("Proton Pump Inhibitors/adverse effects"(Majr) OR "Proton Pump Inhibitors/therapeutic use"(Majr) OR "Proton Pump Inhibitors/toxicity"(Majr)) AND ("Histamine H2 Antagonists/adverse effects"(Majr) OR "Histamine H2 Antagonists/therapeutic use"(Majr) OR "Histamine H2 Antagonists/toxicity"(Majr))

Inclusion and Exclusion Criteria

We included clinical trials, meta-analyses, randomized controlled trials (RCTs), review literature, and a systematic review of full-text articles published in the English language based on humans. There was no limit on the year of publication or age group. We excluded articles such as case reports, expert opinions, animal studies, unpublished gray literature, and articles irrelevant to our research question.

We critically evaluated 35 selected studies for quality using standardized quality assessment tools, and eight studies that were qualified as high or medium quality were included in this group. The following tools were used: (1) for RCTs, the Cochrane risk-of-bias assessment tool; (2) for observational studies, the Newcastle-Ottawa scale; and (3) for traditional reviews, the Scale for the Assessment of Narrative Review Articles (SANRA). Meta-analysis by Scally et al. was reviewed using the Assessment of Multiple Systemic Review (AMSTAR) tool [3].

The detailed overall scores and quality for each study are shown in Tables 1-3 below.

**Table 1:** The Cochrane risk of bias assessment tool shows a low, high, and unclear risk of bias.

Name of the study	Random sequence generation	Allocation concealment	Blinding of participants and personnel	Blinding of outcome assessment	Incomplete outcome data	Selective outcome reporting	Other sources of bias	Remarks
Bardhan et al., 1991 [11]	Low risk	Low risk	Low risk	Low risk	High risk	Low risk	Low risk	6/7, High-quality article
Bate et al., 1989 [12]	Low risk	Low risk	Low risk	Low risk	Low risk	Low risk	Low risk	7/7, High-quality article
Delchier et al., 1989 [13]	Low risk	Low risk	Low risk	Low risk	Risk unclear	Low risk	High risk	5/7, High-quality article
Jones et al., 1997 [14]	Low risk	Low risk	Low risk	High risk	Low risk	High risk	High risk	4/7, Medium-quality article

**Table 2:** The Newcastle-Ottawa Tool shows the quality of the papers.

Study	Selection (max four stars)	Comparability (max two stars)	Outcome (max three stars)	Quality of paper
Lauritsen et al (1988) [15]	***	*	***	High

**Table 3:** The SANRA checklist showing the quality of the papers SANRA: Scale for the Assessment of Narrative Review Articles

Name of the study	Justification of the article's importance for the readership (out of two)	Statement of concrete aims or formulation of questions (out of two)	Description of the literature search (out of two)	Referencing (out of two)	Scientific reasoning (out of two)	Appropriate presentation of data (out of two)	Quality of paper (out of 12)
Tack et al., 2013 [16]	2	2	1	2	2	2	11/12, High quality
Strand et al., 2017 [4]	2	2	2	2	1	2	11/12, High quality

Results

**Figure 1:**
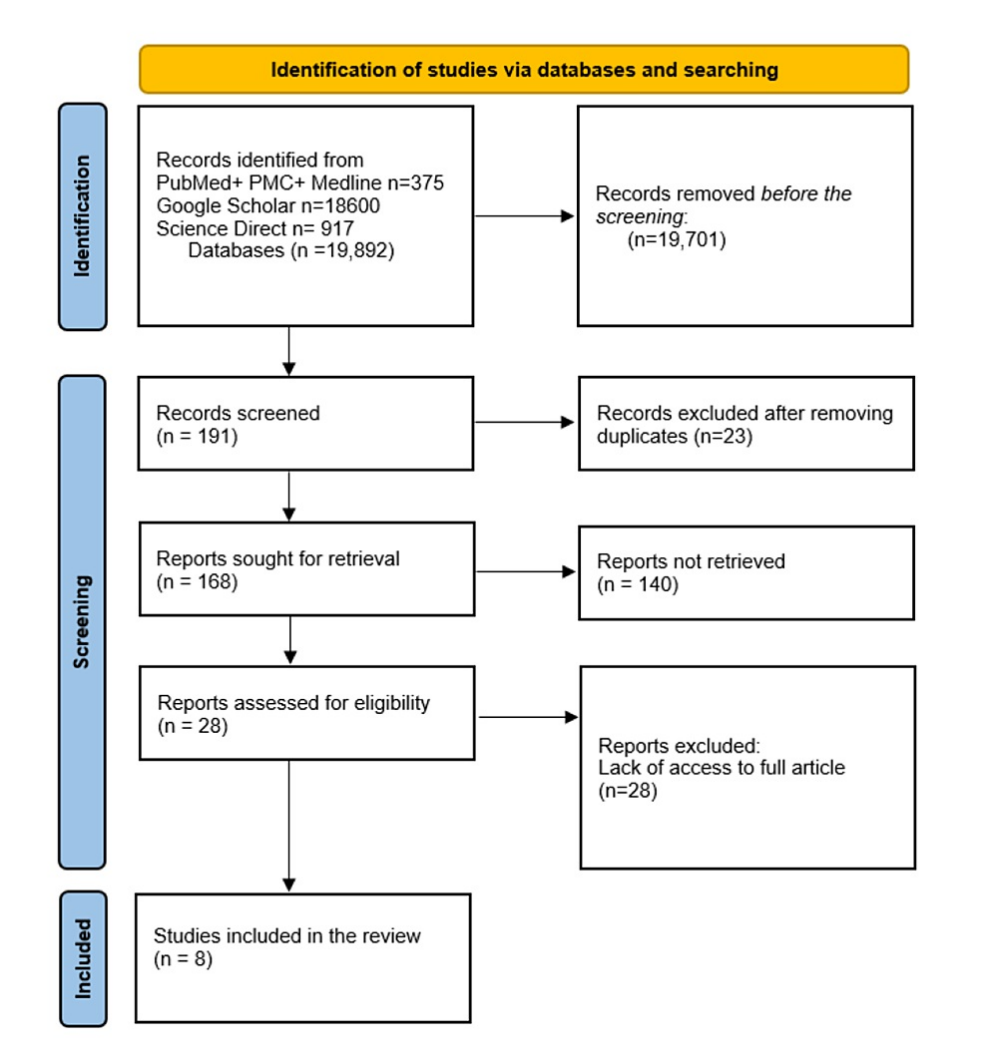
A PRISMA flowchart showcasing the selection of studies PRISMA:  Preferred Reporting Items for Systematic Reviews and Meta-Analyses

A total of 19,892 articles were generated from keywords, eligibility criteria, and databases. Of the total, 375 articles were from PubMed, 18,600 from Google Scholar, and 917 from ScienceDirect. After applying our inclusion criteria, 19,701 articles were removed, leaving 191 to be screened. Duplicates were removed, and 186 articles were screened for their titles and abstracts. A further 140 articles were discarded due to topic irrelevance. Of the remaining 28 articles, eight passed the critical appraisal, as the remaining 20 articles were denied full access to the paper. Figure 1 below shows the selection process in the form of a PRISMA flow chart.

All the reviewed articles differed in design, population, and primary endpoints. However, the effectiveness of PPIs in treating ulcers and their effects on symptoms caused by the ulcer was a standard part of each article. This is shown in a summarized table below in Table 4.

**Table 4:** Medicines, their dosage, and their effect on the various types of ulcers, along with adverse effects PPIs: proton pump inhibitors; H2RAs: histamine-2 receptor antagonists; BD: twice daily; OM: early morning; Mg: magnesium

Name of the author and year of study	Type of study	Dosage of PPIs and H2 receptor blockers	Type of ulcer/ symptom	Distribution of PPIs and H2 receptor blockers among patients	Effectiveness of PPIs and H2 receptor blockers in healing the ulcer	Effectiveness of PPIs and H2 receptor blockers in alleviating epigastric pain	Adverse effects of PPIs and H2 receptor blockers	Conclusion
Lauristen et al.. 1988 [15]	Randomized double-blind comparative trial	Omeprazole 30 mg daily; cimetidine 1g daily	Pre-pyloric gastric ulcer	Omeprazole: 89 patients, cimetidine: 85 patients	Omeprazole: 89% effective after six weeks, cimetidine: 86% effective after six weeks	Omeprazole: 33% of patients cured at four weeks, cimetidine: 32% cured with cimetidine at four weeks	No. of patient complaints with omeprazole: one (headache, fatigue, diarrhea, gastroenteritis, etc.) No. of patient complaints with cimetidine: six (impotence, dizziness, headache, dry mouth)	Omeprazole is superior in healing ulcers and bringing pain relief.
Bate et al., 1989 [12]	Randomized double-blind comparative trial	Omeprazole 20 mg, OM, cimetidine 400 mg, BD	Symptomatic gastric ulcer	Omeprazole: 102 patients, cimetidine: 87 patients	Omeprazole: 84% effective after eight weeks, cimetidine: 75% effective after eight weeks	Omeprazole: 81% of patients were pain-free at four weeks, cimetidine: 60% of patients were pain-free at four weeks	Omeprazole: 19%, cimetidine: 15%	Omeprazole results in faster healing of ulcers and rapid relief of symptoms.
Delchier et al., 1989 [13]	Randomized double-blind comparative trial	Omeprazole 20mg once daily, ranitidine 150 mg twice daily	cimetidine or ranitidine-resistant duodenal ulcer	Omeprazole: 75 patients, ranitidine: 76 patients	Omeprazole: 79.6% effective at four weeks; ranitidine: 75.4% effective at four weeks	Most patients with both drugs were asymptomatic at day 15	Omeprazole: 25%; ranitidine: 29%	¼ patients resistant to empirical treatment of cimetidine or ranitidine recovered with omeprazole 40 mg
Bardhan et al., 1991 [11]	Randomized controlled trial	Omeprazole 40 mg daily, cimetidine 0.8g or 1g daily with ranitidine 0.3g daily	Refractory peptic ulcer	Omeprazole: 54 patients; cimetidine: 35 patients; ranitidine: 18 patients	Omeprazole: 96% of ulcers healed at eight weeks. H2RA blockers: 57% of ulcers healed at eight weeks	Omeprazole: 91% of patients reported relief from epigastric pain. H2RA blockers: 70% reported relief in epigastric pain	Omeprazole: 20%; cimetidine: 34%; ranitidine: 0%	Omeprazole is better than continued treatment with a refractory dose of cimetidine or ranitidine.
Jones et al., 1997 [14]	Randomized double-blind parallel-group study	Lansoprazole 30 mg daily, ranitidine 150 mg BD	Reflux-like/acid-like dyspnea	Lansoprazole: 213 patients; ranitidine: 219 patients	N/A	Omeprazole: 72% of patients reported relief at four weeks. Ranitidine: 60% of patients reported relief at four weeks.	N/A	Lansoprazole works better for heartburn and epigastric pain.
Tack et al., 2013 [16]	Review article	Omeprazole: 20 mg for a gastric ulcer at four to eight weeks; omeprazole for duodenal ulcer: 20 mg for four weeks	Gastric and duodenal ulcers		PPIs are superior to H2RA blockers for healing gastroduodenal ulcers.		Increase the risk of respiratory, GI infection, osteoporosis, and fracture.	Patients with typical reflux symptoms can be started on empirical therapy with PPIs at a standard dose for eight weeks.
Scally et al., 2018 [3]	Meta-analysis of randomized controlled trials		Gastric and duodenal ulcers		PPI was 84% effective in treating gastric and 87% effective in duodenal ulcers. H2RA blockers were 78% effective in gastric and 76% in duodenal ulcers.		PPI is shown to be associated with an increased risk of myocardial infarction [17], bone fracture [18], hypomagnesemia [19], food poisoning and bacterial gut infection [20], dementia [21], and chronic kidney disease [22].	PPIs appeared to be the most effective class of gastroprotection for the management of peptic ulcer disease.
Strand et al., 2017 [4]	Review article	Omeprazole 20 mg daily	Gastric and duodenal ulcers		15.2% therapeutic gain of healing for duodenal ulcers and 9.9% for gastric ulcers for two weeks [23]; P = 0.001 for omeprazole than H2RAs in achieving ulcer healing with an overall rate of 80.8% and 74.7%, respectively [24].	A greater percentage of people were symptom-free with PPIs in the first follow-up.	40% increased dosage of PPIs due to increased nocturnal symptoms [25], low Mg levels [26], community-acquired pneumonia [27], and enteric infection [28].	PPI is very effective in the treatment of acid-related disorders.

Five of the eight chosen articles were randomized, double-blind comparative trials, one meta-analysis, and two review articles. The primary outcome was the effectiveness of PPIs and H2RAs in treating ulcers at various time intervals of four, six, and eight weeks caused by various gastrointestinal (GI) conditions and their effect on epigastric pain. The majority of the articles also compared the adverse effects of both drugs. The population being studied included adult males and females with epigastric pain caused by erosion of the gastric mucosa. Types of ulcers varied from refractory gastric ulcers [11], symptomatic gastric ulcers [12], cimetidine- or ranitidine-resistant duodenal ulcers [13], pre-pyloric gastric ulcers [15], and gastric and duodenal ulcers [3,4,16].

Most studies mentioned the dose and duration required for healing ulcers and symptomatic relief. Before the start of each study, patients were assessed for malignancy associated with an ulcer confirmed by endoscopy [12]. Patients with serious complications such as bleeding and perforation were excluded. Each patient who participated in the trial was asked about the duration, severity, previous drug therapy, and social habits. Patients were given a diary to record the symptoms they experienced in a day. 

In terms of the study outcomes, it is evident in Table 4 that PPIs are better at healing ulcers and causing symptomatic relief; additionally, they are the most effective class of gastroprotection for managing PUD [3]. Some studies showed that H2RA causes more adverse effects than PPIs [11,13,15]. Side effects associated with H2RAs include impotence, dizziness, dry mouth, and headaches [15]. Furthermore, Scally et al. demonstrated [3] the adverse effects of myocardial infarction, bone fracture, hypomagnesemia, bone fracture, community-acquired pneumonia, bacterial gut infection, dementia, and chronic kidney disease in patients using PPIs.

The types of PPIs included in the studies were omeprazole and lansoprazole. Histamine-2 receptor antagonists, such as ranitidine and cimetidine, were used. Drugs, along with their dosage, are mentioned in Table 5.

**Table 5:** Effectiveness of PPI and H2RA at different treatment durations PPI: proton pump inhibitors; H2RA: histamine-2 receptor antagonist; BD: twice daily

Location/Type of ulcer	Dosage of medication	Duration of treatment	Effectiveness in healing the ulcer	Effectiveness in alleviating epigastric pain
Gastric and duodenal ulcer [4,27]	Omeprazole 20 mg daily	Two weeks	Therapeutic gain of 15.2% in healing for duodenal ulcer (p<0.001) and 9.9% for gastric ulcer (p<0.005)	A greater percentage of patients were symptom-free at the first follow-up
H2 receptor-resistant duodenal ulcer [13]	Omeprazole 20 mg once daily, ranitidine 150 mg twice daily	Four weeks	79.6%, 75.4%	No significant difference between the two
Pre-pyloric gastric ulcers [15]	Omeprazole 30 mg daily, cimetidine 1 g daily	Six weeks	89%, 86%	33% at four weeks, 32% at four weeks
Gastric ulcer [12], refractory peptic ulcer [11]	Omeprazole 20 mg once daily, cimetidine 400 mg BD	Eight weeks	84%, 96%	81% at four weeks, 60% at four weeks,

Table 5 compares the effectiveness of PPIs and H2RAs at different treatment durations. Irrespective of the nature of the ulcer, the evidence suggests that PPI results in significantly superior outcomes across all stages. The effectiveness of both drugs in eradicating epigastric pain is also considered. Two studies show a more significant effect of H2RAs on dyspeptic symptoms [11,12]. However, some studies show no significant difference between the two drugs [4, 13].

Discussion

We analyzed the efficacy of PPIs and H2 receptor blockers by comparing both drugs in patients with peptic ulcer disease. Patients had to undergo endoscopy to confirm that the lesion was not associated with any carcinoma. The size of the ulcer was noted before commencing the study, and no other anti-ulcer medication was allowed during the treatment. Patients with severe epigastric pain were not included in the study. Pregnant lactating mothers and patients who had undergone gastric surgery were omitted. Patients taking NSAIDs and anticoagulants were not part of these studies.

Comparing the Effectiveness of Ulcer Healing

According to the study conducted by Bardhan et al. on 107 patients, omeprazole was significantly better than continued H2RA in treating healing refractory peptic ulcers at four and eight weeks [11]. The patients who remained unresponsive to H2RA treatment were treated with an additional four weeks of omeprazole, which increased the cure rate to 86%. The same results were observed in the study conducted by Delchier et al., in which 20 patients with unhealed ulcers were given omeprazole for four weeks, out of which 16 patients were healed [13]. Five out of eight had previously been given omeprazole and 11 of 12 were given ranitidine.

In a randomized double-blinded comparative trial conducted by Bate et al., 74 out of 102 patients (73%) on omeprazole had healed ulcers compared to 50 out of 87 patients (58%) on cimetidine [12]. The therapeutic difference between the percentages is 15%, which moved up to 84% for omeprazole and 75% for cimetidine after eight weeks, moving the therapeutic difference to 9% [12].

A study conducted by Delchier et al. on duodenal ulcers that were resistant to H2RAs (cimetidine ≥ 0.8g, ranitidine ≥300mg daily) despite treatment for six weeks showed a better cure rate with omeprazole (48.3 vs. 46.3%) at two weeks and (79.6% vs. 75.4%) at the end of four weeks [13]. A study carried out by Lauristan et al. on 176 patients with pre-pyloric ulcers consisted of administering 30 mg omeprazole once daily and cimetidine 1 g four times a day; the accumulative healing rate was consistently higher in the omeprazole group compared to the cimetidine group [15]. The difference was more pronounced at two weeks, followed by four or six weeks, and was significantly significant in the intention to treat the cohort.

According to a study by Strand et al., acid suppression therapy remains the mainstay of treatment for gastric and duodenal ulcers [4]. The most important factor for healing an ulcer is the maintenance of stomach pH for 18 to 20 hours [29]. A PPI is the most effective inhibitor of gastric acid secretion as it directly blocks the pump, consistently maintaining the gastric pH > four between 15 and 22 hours daily, compared to only four hours by H2RA [30]. It also discusses a meta-analysis that included 30 double-blind prospective trials of omeprazole (20mg) compared to H2RA, which concluded an overall therapeutic gain of 15.2% in the healing of duodenal ulcers (p<0.001) and 9.9% for gastric ulcers (p<0.05) after two weeks of treatment [27]. In an RCT conducted on 195 patients, omeprazole 20 mg given a week significantly reduced the incidence of recurrent duodenal ulcers when compared to placebo from 67% to 23% (p<0.001) [31]. However, it is worth mentioning that continuous use of H2RA is similarly efficient at preventing ulcer recurrence compared to placebo (20%-25% vs. 60%-90%) [32]. Still, PPI is preferred over H2RA in NSAID or *H. pylori*-associated gastric and duodenal ulcers or when dealing with perforation and fibrosis. This is contrary to the study discussed by Tack et al. [16], which states the most productive approach to preventing ulcers related to NSAIDs is to administer misoprostol, an analog of prostaglandin E1. This contradicts the earlier-mentioned information. [33]. A meta-analysis conducted by Scally et al. showed PPIs as the most effective gastroprotective drug for gastroduodenal ulcers, followed by H2RAs and prostaglandin analogs [3].

Effect of Factors on Healing

According to Bardhan et al., neither ulcer size nor alcohol consumption affected healing [11]. However, this was contradicted by Bate et al., who stated that despite the fact that the size of the ulcer, the healing rate was still better with omeprazole [12]. Bardhan et al. drew another conclusion stating that smoker patients on H2RAs healed better than nonsmokers (48% vs. 20%) [11], which was again contradictory to a study conducted by Bate et al. stating that smoker patients on omeprazole healed at a better rate [12]. Lauristal et al., in their study, highlighted no clear demarcation of the healing rate between smokers and nonsmokers [15]. Delchier et al., in their study, highlighted ulcer size to have the most significant impact on healing; larger ulcers had a significantly lower rate than smaller ones (p = 0.04 and p = 0.02) on days 15 and 29, respectively [13]. This finding is consistent with the conclusion drawn by Lauristeal et al., which stated that ulcer size affects healing [15].

Adverse Effects

In a study by Bardhan et al., 20% of patients on omeprazole and 34% on cimetidine reported adverse effects [11]. Disorders reported with omeprazole use were diarrhea (n=3), loose stools (n=3), and constipation (n=1). Patients on cimetidine reported varied adverse effects such as cramps, headaches, orchitis, and loose stools [11]. These results are analogous to the study conducted by Delchier et al., where 25% of patients on omeprazole and 29% of patients on ranitidine complained of symptoms primarily related to the GI tract, such as vomiting, flatulence, belching, and diarrhea [13]. This is supported by a meta-analysis conducted in 2012 consisting of 42 observational studies with over 313,000 patients, suggesting an association between *Clostridioides difficile* *(C. difficile*) and the use of PPIs [34]. The risk of developing a *C. difficile* infection correlates with the extent of gastric acid suppression. This is due to the increased bacterial growth in the stomach, with the degree of risk proportional to the suppression dosage. [35]. In contrast, a study by Bate et al. on gastric ulcers shows central nervous system (CNS) side effects in 19% of patients taking omeprazole and 15% in the cimetidine group [12]. However, lab values such as hemoglobin (Hb), hematocrit (HCT), platelets (Plt), and creatinine (Cr) remained normal throughout the treatment. This contradicts the study of Lauritsen, where the cimetidine group p<0.05 caused an increase in serum creatinine levels [15].

Proton pump inhibitors have been shown to cause an increased risk of fracture, and this effect is studied in the long-term use of PPIs due to reduced calcium absorption leading to bone fracture, as reported by Strand et al. [36]. However, it was later debunked by Kaye et al., as their study showed no association between fracture and chronic use of PPIs [37]. Nevertheless, the United States Food and Drug Administration (FDA) has added a warning of possible fracture risk (hip, waist, and hip) for patients who take a daily dose of PPI for more than one year. If necessary, patients are advised to take calcium as a preventive measure. Initial use of PPI has also been associated with a likelihood of developing community-acquired pneumonia in 27% of patients, as shown by a meta-analysis of eight observational studies [19], as mentioned by a study conducted by Strand et al. [4]. However, this was questioned later by Filion et al., suggesting that symptoms of gastroesophageal reflux syndrome (GERD) may have been misrepresented as symptoms of community-acquired pneumonia [38]. In 2011, the FDA issued a warning based on 61 individual cases stating that chronic PPIs can cause low magnesium (Mg) levels and should be monitored periodically in patients taking prolonged PPIs, as discussed by Strand et al. [26, 4]. However, this is contradicted by Tack et al. [16], who stated that the risk of developing hypomagnesemia is so low that it does not require follow-up [39].

Correct Dose of PPIs

Varied options exist for treating refractory peptic ulcers, such as continuing treatment with an H2RA for a longer duration at a higher dose or combining it with a mucosal protectant. Still, it has been concluded by Bardhan et al. that omeprazole 40 mg daily heals up to 80% of refractory ulcers within four weeks and almost 100% in eight weeks. It efficiently relieves pain and has high drug safety [11]. A study by Delchier et al. shows that omeprazole 40 mg can treat resistant duodenal ulcers and that omeprazole and ranitidine do not heal at standard dosage [13]. Gastric ulcer treatment, as recommended by Bate et al., involves four weeks of treatment with 20 mg of omeprazole once daily and has a cure rate of 70%-80% [12]; however, cimetidine requires at least eight weeks of treatment with continuation to 16 weeks if symptoms persist.

Dyspeptic symptoms can be treated with lansoprazole 30 mg daily, a valuable substitute for H2RA, as an initial treatment for symptomatic relief, providing additional coverage in patients with the known acid-related disorder, as concluded by Jone et al. [14]. According to Tack et al. [16], the correct dosage of PPIs has a very pressing role in suppressing gastric acid secretion. Daily intake should be consumed 30-60 minutes before breakfast and, in a twice-daily regimen, 30-60 minutes before breakfast and before the last meal of the day [16]. Timing is of utmost importance since PPIs have a shorter half-life of 0.5 to two hours, respectively [33]. We could not find the recurrence of symptoms after patients were weaned off PPIs, along with the effect of carefully following the suggested protocol versus not following the advised regimen. We failed to find a correlation between the location of the ulcer and its effect on epigastric pain. We further need to evaluate the effect of chronic use of PPIs on bone marrow density, vitamin B12 levels, and magnesium since the information available is somewhat ambiguous. A further comparison needs to be made between PPIs and newer interventions such as potassium-competitive acid blockers (P-CABS).

Limitations

Our study has a few limitations; hence, the results should be considered. Most importantly, only one meta-analysis was included in the study out of the eight articles we analyzed. This might affect the quality of our results. Second, we only included articles written in English, which might have led to language bias. Third, our research did not have a standard scale for indicating epigastric pain, which may have led to an overestimation or interpretation of pain reported by patients. Fourth, we did not classify epigastric pain as daytime or nighttime, pre- or postprandial. Fifth, the type and location of ulcers varied greatly among patients in the same study. Lastly, we missed critical studies due to the non-availability of free data from various databases.

## Conclusions

In our study, we tried to determine the safety and efficacy of PPI and H2RA in treating ulcers of various types. Based on the papers we reviewed, PPI is the safest and most effective medication for treating PUD; the improved control of acid secretion with omeprazole results in faster healing and rapid symptom relief. Resistant ulcers can be treated by prolonging the standard dosage. It is evident that omeprazole provides an advantage over H2RA or other available therapies and is more effective in varied clinical settings. Some studies presented symptoms such as diarrhea, loose stools, constipation, headache, and GI upset with a *C. difficile* infection. Hence, prescribers should be careful while advising the drug, considering the comorbidities, expected dose, duration, and method of intake (30-60 minutes before breakfast). Patients should be instructed about the alarming symptoms, such as weight loss, anemia, and dysphagia, and immediately referred to a gastroenterologist for further evaluation. They should be advised to supplement their diet with calcium to eliminate any potential risk of osteoporosis. Although PPI remains the mainstay of treatment for PUD, its safety and efficacy should now be compared with newer acid-suppressing drugs such as potassium-competitive acid blockers (P-CABs) and cholecystokinin (CCK2) receptor antagonists.
